# Perirenal fat thickness and liver fat fraction are independent predictors of MetS in adults with overweight and obesity suspected with NAFLD: a retrospective study

**DOI:** 10.1186/s13098-023-01033-w

**Published:** 2023-03-23

**Authors:** Li Wang, Yuning Pan, Xianwang Ye, Yongmeng Zhu, Yandong Lian, Hui Zhang, Miao Xu, Mengxiao Liu, Xinzhong Ruan

**Affiliations:** 1 Department of Radiology, Ningbo First Hospital, No. 59, Liuting Street, Haishu District, Ningbo, Zhejiang 315010 China; 2 Department of Endocrinology, Ningbo First Hospital, No. 59, Liuting Street, Haishu District, Ningbo, Zhejiang 315010 China; 3 MR Collaborations, Siemens healthineers, No.278, Zhouzhu Road, Pudong New District, Shanghai, 200090 China

**Keywords:** Perirenal fat, Intraorgan fat, Metabolic syndrome, Nonalcoholic fatty liver disease

## Abstract

**Background:**

Nonalcoholic fatty liver disease (NAFLD) has a multidirectional relationship with metabolic syndrome (MetS) and used to be considered a hepatic manifestation of MetS. Perirenal fat, as a part of visceral adipose tissue (VAT), was reported to be correlated with MetS components, but data for intraorgan fat are lacking. This study was undertaken to assess the value of peripheral and intraorgan fat to predict MetS in adults with overweight and obesity with suspected NAFLD.

**Methods:**

We studied 134 sequential adults (mean age, 31.5 years; 47% female) with overweight and obesity with suspected NAFLD. All participants underwent abdominal magnetic resonance imaging (MRI) examination. Anthropometric and metabolic parameters and perirenal fat thickness (PRFT), subcutaneous adipose tissue thickness (SATT), liver fat fraction (LFF), pancreas fat fraction (PFF), and lumbar spine fat fraction (LSFF) were collected. MetS was defined according to the International Diabetes Federation (IDF) criteria. Statistical analyses included basic statistics, linear correlation and logistic regression analysis.

**Results:**

A total of 63 adults with MetS and 71 adults with advanced liver steatosis (grades 2 and 3) were included in our study. Patients with MetS had greater PRFT (p = 0.026) and LFF (p < 0.001), as well as greater homeostasis model assessment of insulin resistance (HOMA-IR), alanine transaminase (ALT), aspartate transaminase (AST), and decreased SATT. MetS patients had a higher proportion of advanced steatosis than those without MetS (P < 0.001). The MetS score was associated with PRFT and LFF. Logistic regression analysis showed that the PRFT and LFF were independent predictors of MetS after adjusting for age and sex. A cutoff of 9.15 mm for PRFT and 14.68% for LFF could be predictive of MetS.

**Conclusions:**

This study shows that the absolute cutoff level of 9.15 mm for PRFT and 14.68% for LFF may be clinically important markers for identifying patients who are at high risk of MetS among adults with overweight and obesity with suspected NAFLD, irrespective of sex and age. Moreover, ectopic fat levels in pancreas and lumbar spine are positively associated with PRFT.

**Trial registration:**

Not applicable.

## Background

MetS, variously known as syndrome X, insulin resistance, etc., is defined by WHO as a pathologic condition characterized by abdominal obesity, insulin resistance, hypertension, and hyperlipidemia [1]. The reported prevalence of MetS in China in 2015–2017 among Chinese residents aged 20 years or older was 31.1% [2], which parallels the incidence of obesity. The total cost of the malady including the cost of health care and loss of potential economic activity is enormous. MetS patients have a high risk of cardiovascular diseases, type 2 diabetes, stroke, and other disabilities. The syndrome is now both a public health and a clinical problem, and individuals with MetS need to be identified effectively. The definition of MetS is slightly different according to various organizations. The three most commonly used definitions are the WHO 1999 criterion, National Cholesterol Education Program (NCEP) ATP3 2005 criterion and IDF 2006 criterion [3]. The IDF criterion for Chinese patients is as waist ≥ 90 cm (males) or ≥ 80 cm (females) along with the presence of two or more of the following: (1) Blood glucose ≥ 5.6 mmol/L or diagnosed diabetes; (2) High-density lipoprotein (HDL) cholesterol < 1.0 mmol/L in males, < 1.3 mmol/L in females or drug treatment for low HDL-C; (3) Blood triglycerides ≥ 1.7 mmol/L or drug treatment for elevated triglycerides; or (4) Systolic blood pressure ≥ 130 and/or diastolic ≥ 85 mmHg or drug treatment for hypertension.

NAFLD is one of the most significant comorbidities of obesity and presents a high degree of comorbidity with disorders of MetS, including type 2 diabetes and cardiovascular disease. Their prevalence has increased worldwide and can be characterized as a growing epidemic, increasing along with the incidence of obesity [4, 5]. NAFLD has a multidirectional relationship with MetS and used to be considered the hepatic consequence of MetS [6]. It was reported that the estimated prevalence of NAFLD in China was 23.8% in the early 2000s, and it reached 32.9% in 2018 in parallel with the rising trend of obesity in China [7].

The diagnosis of NAFLD requires more than or equal to 5% hepatic fat accumulation and exclusion of other etiologies of liver disease, such as viral hepatitis, autoimmune liver disease, hemochromatosis, Wilson’s disease, drug-induced liver disease and significant alcohol consumption [8]. There are several noninvasive methods to quantitatively assess liver fat, including ultrasonography (US), controlled attenuation parameter (CAP), computed tomography (CT), hydrogen-1 magnetic resonance spectroscopy (MRS) and magnetic resonance imaging-estimated proton density fat fraction (MRI-PDFF) [9]. MRI-PDFF, regarded as the most accurate quantitative method for measuring liver fat content in clinical practice, was found to be correlated with histologic steatosis grade and provided reasonable accuracy for noninvasive classification of steatosis grades by Tang and colleagues [10, 11]. Moreover, it could also be used quantitatively evaluate the fat content of other organs, such as the pancreas, kidney, spine and muscle, at the same time.

Data from several studies over the past three decades have shown that MetS is more associated with visceral adipose and ectopic fat tissue than with overall and subcutaneous fat mass (SAT) [12–14]. Increasing visceral accumulation above the threshold is associated with decreased insulin sensitivity and cardiovascular risk independent of total body fat [15]. As a part of visceral adipose tissue, the adipose tissue surrounding the kidney, has been reported as an easily reproducible, indirect measurement of visceral fat, is considered a metabolically active tissue, and has been reported to be associated with hypertension [16] and atherosclerosis [17] in adults. Moreover, the accumulation of perirenal fat (PRF) was reported to correlate with MetS features in patients with obesity [18–20]and was also identified as an emerging cardiovascular risk factor. However, there have been few studies on the connection between MRI-measured PRF and intraorgan fat depots.

Therefore, we enrolled adults with overweight and obesity with suspected NAFLD and divided them into the MetS + and MetS-. Then, we investigated excessive fat depots and the intraorgan fat content, including the PRFT, SATT, and fat contents of the liver, pancreas, and lumbar spine, and their relationship with MetS to identify the subgroups of patients at high risk of MetS.

## Methods

### Study population

**Fig. 1:**
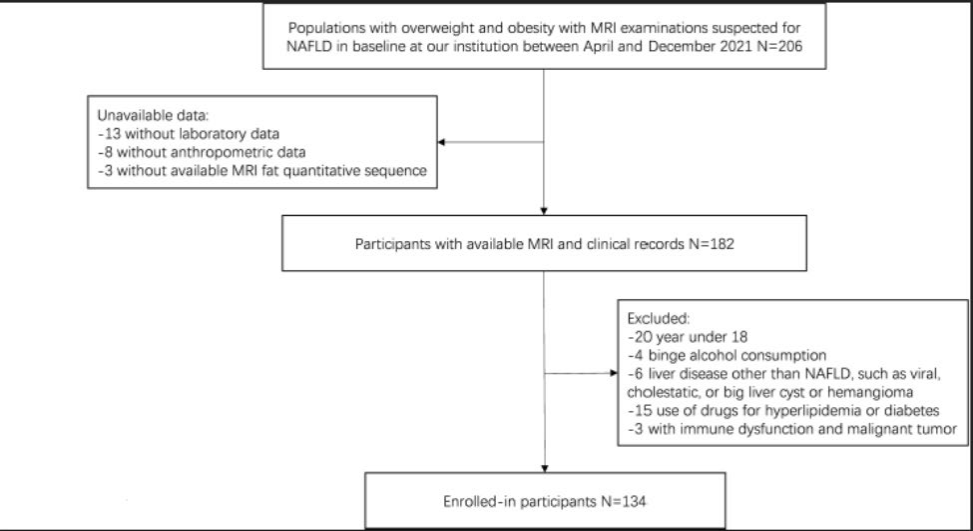
Flowchart of the patient selection and demographics. Populations with overweight and obesity were defined according to World Health Organization BMI cutoffs. Patients were suspected of NAFLD on the basis of clinical, laboratory and US findings. *NAFLD* nonalcoholic fatty liver disease

In this monocentric cross-sectional study, we investigated patients with body mass index (BMI) ≥ 25 kg/m^2^ suspected to have NAFLD on the basis of clinical, laboratory and US data at the Department of Endocrinology, Ningbo First Hospital, Zhejiang, China, from April 2021 to December 2021. Criteria for inclusion were as follows: (1) age ≥ 18 years; (2) evidence of absent or minimal alcohol consumption: <20 g alcohol/day for females and < 30 g alcohol/day for males; (3) absence of confounding disease including acute and/or chronic viral hepatitis (hepatitis A, B, or C); and (4) exclusion of other forms of liver disease including autoimmune, drug-induced, cholestatic and metabolic liver diseases, as well as large liver cysts and hemangioma. The research flowchart is shown in Fig. 1. According to the IDF criteria [3], we divided participants into the MetS + and MetS- groups. The Ethics Committee of Ningbo First Hospital approved the study(022RS). The data are anonymous, and the requirement for informed consent was therefore waived. A total of 134 adults with overweight and obesity with suspected NAFLD were enrolled in the study.

### Clinical and laboratory assessments

All anthropometric parameters were obtained, including age, sex, height, weight, BMI, and waist circumference (WC). Patients were evaluated for all the features of MetS, including diabetes mellitus, hypertension, HDL and central obesity. A WC value ≥ 90 cm in Chinese males and ≥ 80 cm in Chinese females was considered central obesity. AST and ALT, fasting glucose, postprandial blood glucose (PPG), fasting insulin, total cholesterol, HDL cholesterol, LDL-cholesterol, triacylglycerol, uric acid and glycated hemoglobin (HBALC) were measured by our central laboratory. The MetS score is defined as the total number of MetS components present in an individual. Insulin resistance (HOMA-IR) was estimated by the homeostasis model assessment [HOMA-IR = fasting insulin (µU/mL) x fasting glucose (mmol/L)/22.5] [21].

### MRI assessments

The thickness of the SAT and PRF, as well as the MRI-PDFF of visceral organs and the lumbar spine, were measured through a 3.0 T MRI scanner (BioMatrix system, MAGNETOM Vida, Siemens Healthcare, Erlangen, Germany) equipped with an 18-channel array coil. The whole liver and organs in the upper abdomen were covered. The scanning parameters of axial liver acquisition with volume interpolated breath-hold examination (VIBE-Dixon) sequence were as follows: repetition time (TR) = 3.97 ms; echo time (TE) = 1.23 ms; thickness = 3 mm; field of view (FOV) = 420 mm; voxel size = 1.3 mm × 1.3 mm × 3 mm; flip angle = 9°; and averages = 1. The total acquisition time was 13 s. The Q-DIXON sequence of the program named “Liver Quant” was acquired to quantify the fat content of different organs. The parameters of this sequence were TR = 9 ms; TE = 1.05, 2.46, 3.69, 4.92, 6.15, and 7.38 ms; thickness = 3 mm; FOV = 420 mm; voxel size = 1.3 mm × 1.3 mm × 3 mm; flip angle = 4°; and averages = 1. The total acquisition time was 15 s.

**Fig. 2:**
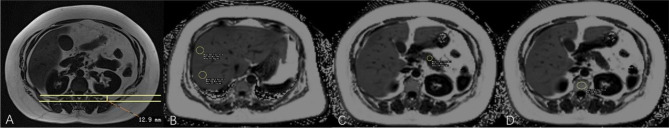
Measurement of PRFT, LFF, PFF and LSFF on MRI map. (A) The assessment of PRFT of a 49 years old femalewith grade 3 NAFLD and MetS on axial MRI fat VIBE-Dixon map. PRFT was calculated by the distance from the anterior margin of the quadratus lumborum muscle to the dorsal margin of the left kidney. (B) The two ROIs in hepatic segments VII-VIII on the PDFF map. LFF was calculated by averaging the results of 8 round ROIs in each of the hepatic segments. (C) ROI in the pancreas body on the PDFF map. The PFF was calculated by averaging the results of three ROIs on the head, body and tail of the pancreas. (D) ROI in the L1 vertebral body on the PDFF map. LSFF measurements were performed by averaging the results of two ROIs on L1-2. *PRFT* perirenal fat thickness, *LFF* liver fat fraction, *PFF* pancreas fat fraction, *LSFF* lumbar spine fat fraction

Patients were instructed to hold their breath during examination. MRI measurement of SAT and PRF was obtained at the level of the exit of the left renal vein, which was easily measured on a transverse section MRI fat VIBE-Dixon image, with the adipose tissue having significantly high signal intensity, while other tissues had significantly low signal intensity (Fig. 2). The SATT was defined as the distance between the skin and external face of the linea alba. The PRFT was defined as the distance from the anterior margin of the quadratus lumborum muscle to the dorsal margin of the left kidney as previously described [22–24].

**Fig. 3:**
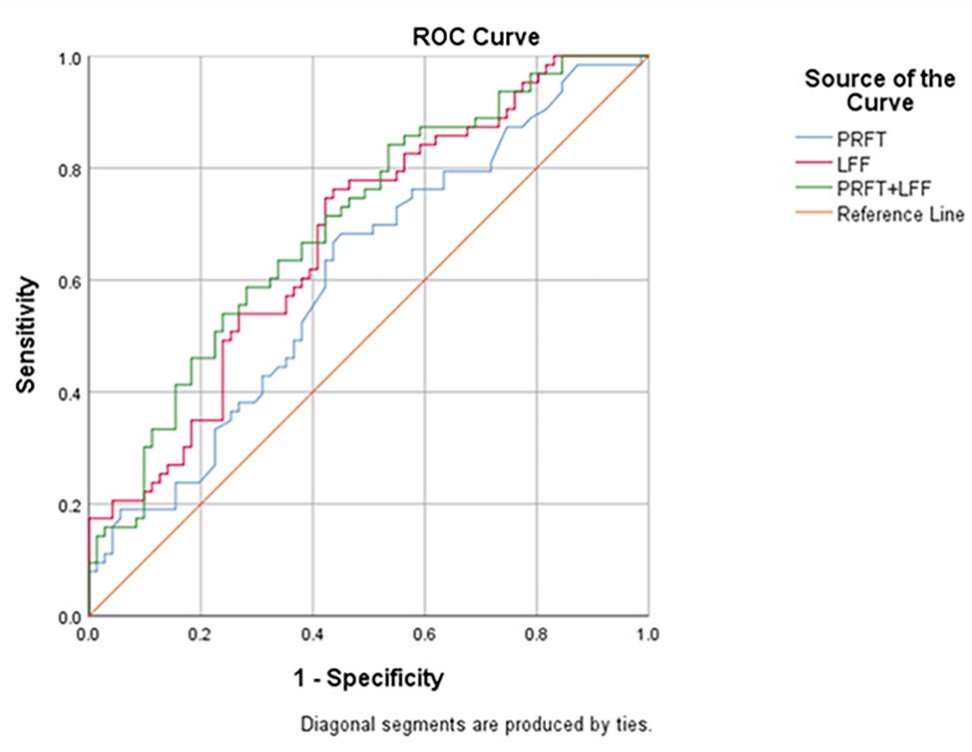
ROC curves for perirenal fat thickness, liver fat fraction and their combination. Y-axis: sensibility; x-axis: 1-specificity. *PRFT* and *LFF* as predictive values for MetS. *PRFT* perirenal fat thickness, *LFF* liver fat fraction, *MetS* metabolic syndrome

Quantitative assessments of the fat contents of the liver, pancreas, and vertebral spine were obtained on the PDFF maps of the Q-Dixon sequence. All MRI images were analyzed by two radiologists with 7 and 5 years of experience. They recorded the LFF by averaging the results of 8 round regions of interest (ROIs) that were more than 2 cm^2^ in each of the hepatic segments on the PDFF map (Fig. 2). The fatty liver grades were defined according to Jens-Peter Kühn’s study as follows: 0, none (PDFF ≤ 5.1%); 1, mild (PDFF > 5.1%); 2, moderate (PDFF > 14.1%); and 3, severe (PDFF > 28.0%) [25]. The PFF was calculated by drawing three ROIs that were more than 1 cm^2^ on the head, body and tail of the pancreas; this process was repeated three times to ensure that all slices showed the pancreas clearly on the postprocessing workstation. LSFF measurements were performed by placing ROIs on the L1-2 vertebral body with more than 2 cm^2^ at the level of the pedicle of the vertebral arch, and the mean PDFF was obtained as the final result by averaging them. All ROIs were placed within the tissue of interested by avoiding major vessels, ducts, and imaging artifacts and ensuring that the ROI was surrounded by the tissue of interest. Image analysis was performed on our hospital patient information system (PACS).

### Statistical analysis

Statistical analysis was performed using SPSS version 25 (IBM Corp, Armonk, Chicago, USA). Continuous variables are expressed as the means (SD); categorical variables are expressed as absolute and relative frequencies. Chi-square tests were used to test for proportions of the categorical variables. Differences between two groups were compared using the Mann‒Whitney U or Student’s t test, as appropriate.

Associations between the MetS score and peripheral and intraorgan fat parameters were studied by Pearson’s and Spearman’s method; logistic regression analysis was used to study independent associations of excessive fat depots, metabolic and anthropometric parameters and MetS after adjustment for potentially confounding factors. The odds ratio (OR) with 95% confidence interval (CI) was determined. The sensitivity and specificity of the PRFT and LFF to predict MetS were assessed using receiver operating characteristic (ROC) curve analysis. Goodness-of-fit was assessed by calculating the area under the curve (AUC), and the optimal cutoff value was determined by the Youden index. A p value < 0.05 was considered to indicate statistical significance.

## Results

### General characteristics, PRFT and intra-organ fat of the whole population

Age ranged from 18 to 59 years old (mean age, 31.5 years). Of a total of 134 adults, 52.99% were male and 47.01% were female. Table 1 shows the baseline characteristics, visceral adipose tissue and intraorgan fat, separated by sex. Sixty-three patients (47.01%) presented with MetS, of whom 33 were male and 30 were female, 15 patients hae fatty liver grade 0 and 1, and 48 patients had grade 2 and 3. Eighty-two patients (61.2%) had advanced liver steatosis (grade 2 and 3), of whom 44 were male and 38 were female. No significant differences in LFF, MetS or advanced liver steatosis were found between males and females. The MetS + group had a higher proportion of advanced liver steatosis. Males showed higher WC, uric acid, PFF, and LSFF and thicker PRFT, whereas females had significantly larger HDL and thicker SATT (p < 0.05). The PRFT was significantly greater in males than in females (15.19 ± 7.06 mm vs. 7.72 ± 5.38 mm, P < 0.001).

**Table 1:** Clinical features of all participants according to sex

Characteristics	Overall (n = 134)	Male(n = 71)	Female (n = 63)	*p* value
Age (y)	31.5 (9.16)	31.49 (9.90)	31.51 (8.34)	0.993
BMI (kg/m^2^)	33.96 (4.75)	34.25 (4.37)	33.63 (5.16)	0.451
Waist circumference (cm)	106.84 (11.5)	110.7 (10.03)	102.51 (11.57)	< 0.001
Fasting glucose (mmol/L)	5.77(1.62)	5.58(1.21)	5.99(1.98)	0.142
Systolic blood pressure (mmHg)	134.27(14.93)	135.38(13.08)	133.02(16.79)	0.362
Diastolic blood pressure (mmHg)	82.48(10.95)	82.82 (10.52)	82.10(11.50)	0.705
Uric acid (µmol/L)	448.33 (108.13)	500.2 (106.21)	390.69 (76.98)	< 0.001
Triglycerides (mmol/L)	2.19 (2.77)	2.57 (3.70)	1.76 (0.78)	0.087
HDL (mmol/L)	1.12 (0.22)	1.07 (0.23)	1.18 (0.20)	0.002
LDL (mmol/L)	3.7 (0.94)	3.64 (0.93)	3.78 (0.95)	0.385
ALT (U/L)	70.58 (54.66)	77.62 (51.41)	62.65 (57.48)	0.114
AST (U/L)	43.23(32.22)	42.45(24.74)	44.11(39.18)	0.773
HOMA-IR	8.37 (5.96)	8.57 (6.32)	8.15 (5.57)	0.689
PRFT (mm)	11.68 (7.33)	15.19 (7.06)	7.72 (5.39)	< 0.001
SATT (mm)	29.47 (10.23)	25.36 (9.23)	34.1 (9.33)	< 0.001
LFF (%)	17.25 (10.19)	16.88 (9.33)	17.66 (11.15)	0.662
PFF (%)	5.9 (5.35)	7.14 (6.07)	4.45 (4.01)	0.004
LSFF (%)	41.43 (9.7)	44.11 (9.22)	38.41 (9.40)	0.001
MetS (%)	47.01	46.48	47.62	0.895

Data are presented as mean (SD) or percentage. Results were based on analyses weighted towards the sex distribution of the general population.

*HDL* high density lipoprotein, *LDL* low density lipoprotein, *ALT* alanine transaminase, *AST* aspartate transaminase, *HOMA-IR* homeostasis model assessment of insulin resistance, *PRFT* perirenal fat thickness, *SATT* subcutaneous adipose tissue thickness, *LFF* liver fat fraction, *PFF* pancreas fat fraction, *LSFF* lumbar spine fat fraction, *MetS* metabolic syndrome.

### The clinical and biochemical characteristics and excessive and intraorgan fat depots according to MetS and fatty liver grade

The MetS + group had a higher proportion of advanced steatosis (58.54% vs. 28.85%, P < 0.001) and higher LFF (20.7 ± 10.5% vs. 14.18 ± 8.9%, P < 0.001) than those of the MetS- group. In comparison with those of the MetS- group, the PRFT of the MetS + group was significantly increased (13.17 ± 7.7 mm vs. 10.35 ± 6.78 mm, *P* < 0.05), whereas the SATT was significantly decreased (27.53 ± 10.07 mm vs. 31.19 ± 10.12 mm, *P* < 0.05) (Table 2). The MetS + group had higher HOMA-IR, ALT and AST levels. Patients with advanced liver steatosis (grade 2 and 3) had greater PRFT than those with grade 0 and 1 fatty liver (12.79 ± 7.60 mm vs. 9.92 ± 6.58 mm, *P* < 0.05), as well as higher WC, fasting glucose, triglycerides, HOMA-IR, ALT, AST, and glycated hemoglobin (Table 3).

**Table 2:** Clinical and biochemical characteristics and ectopic fat depots according to MetS

Characteristics	n	Patients with MetS(n = 63)	Patients without MetS(n = 71)	*p* value
PRFT (mm)	134	13.17 (7.70)	10.35 (6.78)	0.026
SATT (mm)	134	27.53 (10.07)	31.19 (10.12)	0.038
Age (y)	134	33.06(9.20)	30.11(8.97)	0.063
BMI (kg/m^2^)	134	33.77(4.56)	34.12(4.93)	0.668
Waist circumference (cm)	134	107.46(10.86)	106.30(12.09)	0.584
Fasting insulin (pmol/L)	134	236.80(127.52)	207.23 (117.51)	0.165
HOMA-IR	134	9.81 (6.86)	7.09 (4.72)	0.009
ALT (U/L)	134	84.27 (60.72)	58.44 (45.74)	0.006
AST (U/L)	134	51.00 (36.46)	36.34 (26.32)	0.008
LFF (%)	134	20.70(10.50)	14.18 (8.92)	< 0.001
PFF (%)	133	6.67 (6.25)	5.20(4.39)	0.114
Pancreas head fat fraction (%)	133	4.02(3.44)	3.05(3.06)	0.086
Pancreas body fat fraction (%)	133	5.96(7.20)	4.51(4.31)	0.157
Pancreas tail fat fraction (%)	133	10.05(10.65)	8.03(8.0)	0.217
LSFF (%)	134	42.12(9.82)	40.82 (9.62)	0.440

Data are presented as mean (SD) or percentage. Results were based on analyses according to the MetS of the general population.

*MetS* metabolic syndrome, *PRFT* perirenal fat thickness, *SATT* subcutaneous adipose tissue thickness, *HOMA-IR* homeostasis model assessment of insulin resistance, *ALT* alanine transaminase, *AST* aspartate transaminase, *LFF* liver fat fraction, *PFF* pancreas fat fraction, *LSFF* lumbar spine fat fraction.

**Table 3:** The differences in clinical, biochemical characteristics, subcutaneous fat, perirenal fat thickness and intraorgan fat depots between fatty liver grade 0 & 1 and grade 2 & 3

Characteristics	Grade 0 & 1(n = 52)	Grade 2 & 3(n = 82)	*P* value
Age (y)	30.75 (9.03)	31.98 (9.26)	0.453
BMI (kg/m^2^)	33.5 (4.18)	34.25 (5.08)	0.354
Waist circumference (cm)	104.04 (10.62)	108.62 (11.74)	0.024
Fasting glucose (mmol/L)	5.31 (0.63)	6.06 (1.97)	0.002
Uric acid (µmol/L)	432.18 (101.05)	459.00 (111.58)	0.15
Triglycerides (mmol/L)	1.59 (0.93)	2.57 (3.41)	0.045
HDL (mmol/L)	1.15 (0.22)	1.10 (0.23)	0.287
ALT (U/L)	38.33 (28.08)	91.04 (57.60)	<0.001
AST (U/L)	26.31 (15.66)	53.96 (35.36)	<0.001
Fasting insulin (pmol/L)	198.79 (142.45)	235.99 (107.44)	0.089
HOMA-IR	6.92 (5.83)	9.29 (5.89)	0.024
HbAlc (%)	5.37 (0.40)	6.19 (1.39)	<0.001
PRFT (mm)	9.92 (6.58)	12.79 (7.60)	0.027
SATT (mm)	30.09 (9.00)	29.07 (10.97)	0.577
PFF (%)	5.97 (4.78)	5.85 (5.71)	0.907
LSFF (%)	40.65 (11.19)	41.92 (8.66)	0.489
MetS (%)	28.85	58.54	< 0.001

Data are presented as mean (SD) or percentage. Results were based on analyses weighted towards the fatty liver grade of the general population

*HDL* high density lipoprotein, *ALT* alanine transaminase, *AST* aspartate transaminase, *HOMA-IR* homeostasis model assessment of insulin resistance, *HbAlc* glycosylated hemoglobin, *PRFT* perirenal fat thickness, *SATT* subcutaneous adipose tissue thickness, *PFF* pancreas fat fraction, *LSFF* lumbar spine fat fraction, *MetS* metabolic syndrome

### The relationships of anthropometric and biochemical parametersand peripheral and intraorgan fat depots with MetS

We observed a significant positive correlation between PRFT and age (r = 0.297; p < 0.0001), BMI (r = 0.244; p = 0.004), WC (r = 0.402; p < 0.0001), uric acid (r = 0.315; p < 0.0001), ALT (r = 0.176; p = 0.041), fasting insulin (r = 0.182; p = 0.036), PFF (r = 0.314; p < 0.0001), and LSFF (r = 0.225; p = 0.009), whereas PRFT showed a negative correlation with SATT (r=-0.339; p < 0.0001), and HDL (r=-0.248; p = 0.004). There were no significant correlations between PRFT and LFF (r = 0.077; p = 0.378), fatty liver grade (r = 0.147; p = 0.090), and HOMA-IR (r = 0.167; p = 0.055). Fatty liver grade was positively correlated with fasting glucose (r = 0.306; p < 0.0001), fasting insulin (r = 0.251; p = 0.003), HOMA-IR (r = 0.331; p < 0.0001), ALT (r = 0.584; p < 0.0001), AST (r = 0.576; p < 0.0001), and uric acid (r = 0.197; p = 0.023). With regard to intraorgan fat depots, PFF showed a significant positive correlation with PRFT, age, and LSFF and was negatively correlated with SATT, and LSFF was positively correlated with PRFT, age, BMI, WHR, uric acid, and PFF and negatively correlated with SATT.

Univariable analysis showed that PRFT, HOMA-IR, ALT, AST and LFF in patients with MetS were significantly increased compared with those in patients without MetS, while SATT was significantly decreased in patients with MetS (Table 3). In Table 4, the association of the MetS score, ranging from zero to five, with peripheral and intraorgan fat parameters is shown. PRFT (p = 0.013) and LFF (p < 0.001) were significantly associated with the MetS score. The association between biochemical parameters, intraorgan fat, excessive fat depots and MetS was assessed by using logistic regression analysis. The results showed that the PRFT and LFF were significant and independent predictors for the presence of MetS (OR = 1.061, 95% CI, 1.007–1.118; p = 0.026; and OR = 1.077; 95% CI, 1.035–1.121; p < 0.001) after adjusting for confounding factors, i.e., age and sex, including the PRFT, SATT, HOMA-IR, ALT, AST, LFF, and pancreas head fat fraction as predictive values with MetS as the dependent value.

**Table 4:** Relationship of peripheral and intraorgan fat parameters to metabolic syndrome scores (range 0–5)

Peripheral and intra-organ fat parameters	r	*p* value
Waist circumference	0.521	0.056
SATT	-0.161	0.063
PRFT	0.215	0.013
LFF	0.324	< 0.001
PFF	0.1	0.252
LSFF	0.024	0.784

*SATT* subcutaneous adipose tissue thickness, *PRFT* perirenal fat thickness, *LFF* liver fat fraction, *PFF* pancreas fat fraction, *LSFF* lumbar spine fat fraction.

### Predictive analysis

ROC curves analysis showed a cutoff point for PRFT of 9.15 mm to predict MetS with a sensitivity of 0.683 and specificity of 0.549 (AUC = 0.610, p = 0.028), and a cutoff point for LFF of 14.68% to predict MetS with a sensitivity of 0.762 and specificity of 0.563 (AUC = 0.679, p < 0.001). Moreover, the combination of PRFT and LFF better predicted MetS with a sensitivity of 0.841 and specificity of 0.465 (AUC = 0.70 and p < 0.001) (Fig. 3).

## Discussion

In our study, we used PRFT as an easily reproducible method to measure visceral fat indirectly on MRI. Univariable analysis revealed that PRFT, SATT, HOMA-IR, ALT, AST and LFF were associated with MetS. Adjusting for many potential confounding variables, logistic regression analysis showed that PRFT and LFF were independent predictors of the presence of MetS in adults with overweight and obesity suspected of having NAFLD. ROC analysis showed the cutoff point for PRFT of 9.15 mm was an important indicator of MetS in our study. Previous studies showed that PRF as visceral fat was strongly associated with diastolic blood pressure level [16], the risk of developing of chronic kidney disease in diabetes [26], metabolic risk factors in patients with chronic kidney disease [27], and postoperative complications after laparoscopic distal gastrectomy for gastric cancer [24]. Anatomy studies have proven that perirenal fat is unique compared to other connective tissues in that it is well vascularized, innervated, and drains into the lymphatic system [28–30]. In Liu’s study, excessive perirenal adiposity increased the risk of coronary heart disease (CHD) and hypertension through adipokine secretion, fat–kidney interactions, and the neural reflex [31]. A recent study also showed that a cutoff of 22.5 mm (M)/12.5 mm (F) of perirenal fat measured by ultrasound could be predictive of later MetS onset [19]. Further studies are needed to evaluate the value of these cutoff measures as prognostic markers in larger studies.

Data from recent studies showed that the MRI measured liver fat content was significantly associated with an increased risk of MetS, and an increase in the amount of liver fat had a clear dose-response relationship with the presence of MetS and the number of MetS components among adults [32, 33]. NAFLD used to be considered the hepatic consequence of MetS. In this study, our results showed that LFF measured by MRI-PDFF was an independent predictor for the presence of MetS, even after adjusting for age and sex. We proposed a cutoff level of 14.68% for LFF may be a clinically important marker for identifying NAFLD patients who are at high risk of MetS. ROC analysis showed that compared with PRFT, the area under the curve of LFF was larger. Furthermore, the combination of PRFT and LFF better predicted MetS with a sensitivity of 0.841, specificity of 0.465, and AUC = 0.70.

In this study, another significant correlation between the PRFT and intraorgan fat content has also been described, that is, the LFF, PFF and LSFF. To the best of our knowledge, this study, performed in Chinese adults with overweight and obesity suspected to have NAFLD, is the first to show a direct relationship between perirenal fat and intraorgan fat depots, such as the liver, pancreas and lumbar spine, measured by MRI. These results suggest that the perirenal fat and intraliver, intrapancreas and intralumbar spine fat depots were simultaneously increased in overweight or obese individuals suspected of having NAFLD, supporting the coexistence of excessive fat depots and intraorgan fat depots in such patients. In line with this hypothesis, our results agreed with the study of Cuatrecasas et al. [19] that patients with advanced liver steatosis showed larger amounts of perirenal fat compared to that of patients with fatty liver grade 0 and 1. Linear correlation analysis showed that there was no significant correlation between PRFT and LFF or liver steatosis grades. Further research with a large sample size is needed to examine the correlations between excessive fat depots and ectopic fat depots to identify the organs that are vulnerable to MetS.

With regard to the positive correlation between PRFT and PFF, as well as fasting insulin, our results agreed with those of previous studies that PRFT was independently associated with higher insulin resistance [34], and fatty pancreas was correlated with insulin resistance and β-cell dysfunction [35–38]. A study by Nadarajah and colleagues showed that there was a significant difference between the T2DM and control groups with respect to the fat fraction in the pancreatic head, body, and tail, and increased fat in the pancreatic tail may identify patients at risk of T2DM [39], while in our study, the value of the pancreas fat content in identifying MetS was not found. Few studies have investigated the relationship between PRFT and LSFF, but our results partly agreed with Bredella et al. [40] that ectopic fat was positively associated with bone marrow fat in adults with obesity. Further research is needed to elucidate the mechanisms linking these excessive visceral fat depots with intraorgan fat depots.

US has been widely used to measure visceral fat to find the association between abdominal fat layers and MetS features in a number of studies [16, 19, 20, 27]. Nevertheless, the evaluation is subjective and relies on the operator’s experience. Quantitative analysis of PRFT by MRI has a higher degree of precision and reproducibility than US [41]. Furthermore, PRFT/SATT and MRI-PDFF of different organs can be precisely quantified within 1 min. Thus, it is a promising, more accurate and reproducible method preferred over US. Controversy exists regarding the reference single axial slice that is used to assess VAT/SAT at baseline and predict changes. In our study, the level of exit of the left renal vein was near the level of the L1–L2 intervertebral disc, which was considered the optimized site that can be used for quantitating the amount of VAT/SAT on MR examinations [42], and the optimal site for assessing perirenal fat was near the central level of the left kidney. Since the assessment of fat distribution of VAT in clinical practice remains a challenge, PRFT and LFF measured by MRI may be a simple, convenient, easy-to-reproduce, clinically applicable tool to monitor changes in VAT and ectopic fat, implying their role as emerging MetS and cardiovascular risk factors.

Our study had several limitations. First, the data were derived from our single-center institution, and our sample size was relatively small and thus cannot represent the current state of the whole country. Second, it was an observational cross-sectional design, and longitudinal data and outcomes were lacking. Third, the causal effect of the association between PRFT and MetS cannot be elucidated. Finally, the cutoff value as a prognostic marker should be evaluated in larger studies.

## Conclusions

The present study, performed in adults with overweight and obesity with suspected NAFLD, showed that the absolute cutoff level of 9.15 mm for PRFT and 14.68% for LFF may be clinically important markers for identifying patients who are at high risk of MetS, irrespective of sex and age. Moreover, ectopic fat levels in the pancreas and lumbar spines are positively associated with PRFT.

## Data Availability

The data and material that support the findings of this study are available from the corresponding author on reasonable request.

## Abbreviations

ALT: Alanine transaminase
AST: Aspartate transaminase
AUC: area under the curve
BMI: Body mass index
CAP: Controlled attenuation parameter
CT: Computed tomography
CI: Confidence interval
CHD: Coronary heart disease
FOV: Field of view
HDL: High-density lipoprotein
HBALC: Glycated hemoglobin
HOMA-IR: Homeostasis model assessment of insulin resistance
IDF: International Diabetes Federation
LFF: Liver fat fraction
LSFF: Lumbar spines fat fraction
MetS: Metabolic syndrome
MRI: Magnetic resonance imaging
MRS: Magnetic resonance spectroscopy
MRI-PDFF: Magnetic resonance imaging-estimated proton density fat fraction
NAFLD: Nonalcoholic fatty liver disease
NCEP: National Cholesterol Education Program
OR: Odds ratio
PFF: Pancreas fat fraction
PRF: Perirenal fat
PRFT: Perirenal fat thickness
ROC: Receiver operating characteristic
ROI: Regions of interest
SAT: Subcutaneous fat mass
SATT: Subcutaneous adipose tissue thickness
TR: Repetition time
TE: Echo time
US: Ultrasonography
VAT: Visceral adipose tissue
VIBE: Volume interpolated breath-hold examination
WC: Waist circumference.
